# OX40L-Armed Oncolytic Virus Boosts T-cell Response and Remodels Tumor Microenvironment for Pancreatic Cancer Treatment

**DOI:** 10.7150/thno.83495

**Published:** 2023-07-09

**Authors:** Shiyu Liu, Fan Li, Qiongqiong Ma, Mingjuan Du, Haoran Wang, Yiping Zhu, Li Deng, Wenrui Gao, Chunlei Wang, Yanqin Liu, Zhuoqian Zhao, Huanzhen Liu, Ruikun Wang, Yujie Tian, Manli Hu, Yajuan Wan, Wenyi Lu, Meng Zhang, Mingfeng Zhao, Youjia Cao, Hongkai Zhang, Wei Wang, Hui Wang, Yuan Wang

**Affiliations:** 1 State Key Laboratory of Medicinal Chemical Biology and College of life science, Nankai University, Tianjin, China.; 2 CNBG-Nankai University Joint Research and Development Center, Tianjin, China.; 3 Shanghai Institute for Advanced Immunochemical Studies, ShanghaiTech University, Shanghai, China.; 4 Department of Hematology, Tianjin First Central Hospital, Tianjin 300192, China.

**Keywords:** oncolytic virus, pancreatic cancer, OX40L, tumor immune microenvironment, T cell

## Abstract

**Rationale:** The resistance of pancreatic ductal adenocarcinoma (PDAC) to immunotherapies is caused by the immunosuppressive tumor microenvironment (TME) and dense extracellular matrix. Currently, the efficacy of an isolated strategy targeting stromal desmoplasia or immune cells has been met with limited success in the treatment of pancreatic cancer. Oncolytic virus (OV) therapy can remodel the TME and damage tumor cells either by directly killing them or by enhancing the anti-tumor immune response, which holds promise for the treatment of PDAC. This study aimed to investigate the therapeutic effect of OX40L-armed OV on PDAC and to elucidate the underlying mechanisms.

**Methods:** Murine OX40L was inserted into herpes simplex virus-1 (HSV-1) to construct OV-mOX40L. Its expression and function were assessed using reporter cells, cytopathic effect, and immunogenic cell death assays. The efficacy of OV-mOX40L was then evaluated in a KPC syngeneic mouse model. Tumor-infiltrating immune and stromal cells were analyzed using flow cytometry and single-cell RNA sequencing to gain insight into the mechanisms of oncolytic virotherapy.

**Results:** OV-mOX40L treatment delayed tumor growth in KPC tumor-bearing C57BL/6 mice. It also boosted the tumor-infiltrating CD4+ T cell response, mitigated cytotoxic T lymphocyte (CTL) exhaustion, and reduced the number of regulatory T cells. The treatment of OV-mOX40L reprogrammed macrophages and neutrophils to a more pro-inflammatory anti-tumor state. In addition, the number of myofibroblastic cancer-associated fibroblasts (CAF) was reduced after treatment. Based on single-cell sequencing analysis, OV-mOX40L, in combination with anti-IL6 and anti-PD-1, significantly extended the lifespan of PDAC mice.

**Conclusion:** OV-mOX40L converted the immunosuppressive tumor immune microenvironment to a more activated state, remodeled the stromal matrix, and enhanced T cell response. OV-mOX40L significantly prolonged the survival of PDAC mice, either as a monotherapy or in combination with synergistic antibodies. Thus, this study provides a multimodal therapeutic strategy for pancreatic cancer treatment.

## Introduction

Pancreatic ductal adenocarcinoma (PDAC) is one of the most lethal solid tumors, with 466,003 deaths worldwide in 2020 1, 2. Despite advances in conventional chemotherapy and radiation, they have not been shown to significantly improve life expectancy in patients with PDAC. Similarly, immunotherapy has yet to be proven effective 3. Resistance to immunotherapies in PDAC is caused by immunosuppressive tumor microenvironment (TME) and dense extracellular matrix 4.

Recent discoveries in immunology and the TME have inspired the development of oncolytic virotherapy 5-7. Oncolytic viruses (OVs) have the ability to convert immunologically “cold” tumors into “hot” ones by activating both innate and adaptive immunity 8, 9. Talimogene laherparepvec (T-VEC), the first FDA-approved OV that was a granulocyte-macrophage colony-stimulating factor (GM-CSF)-armed herpes simplex virus-1 (HSV-1), could effectively reduce the tumor burden in both injected and non-injected melanoma lesions 10-13. Previous research reported that oncolytic HSV-1 reprogrammed immunosuppressive TME to a more proinflammatory state, especially decreased anti-inflammatory macrophages 9. In addition, CD40L-expressing HSV-1 therapy promoted dendritic cell (DC) maturation and cytotoxic T cell activation, significantly prolonging the survival of mice with PDAC 14. Intratumoral delivery of transgenes through OV enables gene expression* in situ*, which is crucial for some immune-stimulating proteins because the systemic administration of these immune modulators is associated with severe adverse effects or weak immune responses at the tumor location.

OX40 belongs to the tumor necrosis factor receptor superfamily (TNFRSF) and is widely expressed on activated T cells and neutrophils. The interactions between OX40 and OX40L act as co-stimulatory signals for T-cell activation that enhance the proliferation, survival, activation, and differentiation of CD4+ and CD8+ T cells and block the suppressive function of regulatory T cells (Tregs) 15, 16. Furthermore, OX40L and OX40 agonists promote the activation and survival of neutrophils 17, 18. Nevertheless, OX40 agonist therapy in pancreatic cancer is hindered by inefficient T cell trafficking into tumor immunosuppressive TME. DNX-2440, an OX40 ligand expressing oncolytic adenovirus, triggered an anti-tumor immune response and led to prolonged survival in preclinical models of cancers such as gliomas 19. In addition, virus-like particles (VLPs) harboring OX40 ligand showed potential for boosting T-cell activation and eliminating tumor cells 20. Nevertheless, HSV-1 has its own advantages for its bigger capability to insert multiple transgenes and more potent oncolytic activity. Furthermore, two HSV-1-derived virotherapy have been approved for marketing, and the efficacy and safety of HSV-1-based oncolytic therapies have been clinically validated 21-23.

Hence, we engineered an HSV-1-based murine OX40L-expressing oncolytic virus (OV-mOX40L). OV-mOX40L exhibited local and systemic tumor suppression and prolonged the survival of PDAC mice. Single-cell RNA sequencing (scRNA-seq) revealed that OV-mOX40L treatment activated tumor-infiltrating CD4 and CD8 T cells and decreased Tregs, along with the reprogramming of macrophages and neutrophils to a more pro-inflammatory anti-tumor state. Moreover, OV-mOX40L remodeled the stromal matrix to promote T-cell infiltration. This study aimed to investigate the immunological effects of OV-mOX40L on tumor-infiltrating immune cells, which could potentially be used as monotherapy or in combination with synergistic antibodies for PDAC treatment.

## Materials and Methods

### Cells

Vero cells were originally obtained from ATCC (CCL-81, ATCC). KPC cell line was purchased from Shanghai Biomodel Organism Science & Technology Development Co., Ltd, (Catalog number: NM-YD04). KPC mice were obtained by crossing Trp53-R172H, Kras-LSL-G12D with Pdx1-Cre-Tg. The KPC mouse contains a dominant negative point mutation (Trp53-R172H) in the *P53* gene and a conditionally activating point mutation (Kras-LSL-G12D) in the *KRAS* gene with a lox-stop-lox (LSL) termination sequence upstream. Cre-mediated recombination excises the LSL termination sequences and enables expression of KRASG12D in pancreatic tissue 24. KPC cells derived from the KPC mice and were capable of tumor formation after subcutaneous inoculation in C57BL/6 mice. These cells were cultured in DMEM supplemented with 10% fetal bovine serum (FBS) and 1% penicillin/streptomycin and incubated at 37 °C with 5% CO_2_.

### Construction of oncolytic viruses

The trimerized membrane-bound OX40L was constructed by linking a mouse TRAF2 coiled-coil domain to a mouse OX40L extracellular domain. An interleukin-2 (IL-2) signal sequence and a PDGFR transmembrane domain were added at the 5' and 3' ends, respectively. The expression cassettes were inserted into the ICP34.5 location of the HSV-1 genome to construct OV-mOX40L. The viruses were propagated, titered, and purified as previously described 9.

### Immunogenic cell death analysis

Approximately 2×10^5^ cells were mixed with the oncolytic virus at a multiplicity of infection (MOI) of 1. After 48 hours, the supernatant was collected to detect the ATP concentration using the Enhanced ATP Assay Kit (S0027, Beyotime). The tumor cells were stained with anti-calreticulin (CRT) antibody (bs-5913R-AF647, Bioss), and subjected to flow cytometry analysis.

### Reporter cells assay

Jurkat-NF-κB-GFP-OX40 reporter cells were constructed to detect the function of OV-mOX40L. Jurkat cells were stably transfected with a GFP reporter gene under the control of NF-κB response element and OX40 sequences. Approximately 1×10^6^ HEK293FT cells were mixed with the oncolytic virus at an MOI of 1. Twenty-four hours after infection, the infected HEK293FT cells were mixed with 5×10^6^ Jurkat-NF-κB-GFP-OX40 reporter cells for 24 hours of coculture. The reporter Jurkat cells were harvested and subjected to flow cytometry analysis to detect the expression of GFP.

### *Ex vivo* assay

Approximately 1×10^5^ KPC cells were mixed with an oncolytic virus at an MOI of 1 and incubated for 24 hours. Mouse spleen was separated from C57BL6/J mice and ground into splenocytes, then CD3+ T cells or Tregs were sorted by magnetic beads (130-094-973, 130-095-925, Miltenyi), respectively. The cell culture medium of KPC infected with the oncolytic virus was replaced with RPMI1640 complete medium (RPMI1640+10% FBS+2% penicillin/streptomycin) and added 5×10^5^ isolated T cells or Tregs, cocultured for 24 hours. Then T cells were harvested and stained with CD4, CD8, IFN-γ, and granzyme B antibodies, and subjected to flow cytometry analysis. The Treg cells were harvested and subjected to RNA extraction and reverse transcription. Foxp3 expression was detected by quantitative real-time PCR. For T cell proliferation, 5×10^5^ isolated CD3+ T cells were labeled with carboxyfluorescein succinimidyl ester (CFSE) and cocultured with 1×10^5^ OV-infected KPC cells in a 96-well plate precoated with anti-CD3 antibody for three days. The proliferation of T cells was determined by flow cytometry.

### Mice model

The C57BL/6J mice were obtained from the Vital River Laboratory Animal Technology (Beijing, PR China). All animal studies were performed in accordance with the Institute Research Ethics Committee of Nankai University (Protocol Registry Number: A-2018-0306).

For the KPC syngeneic tumor mouse model, approximately 5×10^6^ KPC cells were mixed with Matrigel (v:v=1:1) and subcutaneously implanted into the right flank of 6-week-old male mice. Once the tumor volumes reached 100-150 mm^3^ (day 0), the tumor-bearing mice were treated intratumorally with 2×10^6^ PFU of purified oncolytic virus per mouse on days 0, 3, 6, 9, and 12. For the combination therapy, anti-IL6 (BE0046, Bioxcell) and anti-PD-1 (BE0146, Bioxcell) neutralizing antibodies were injected intraperitoneally on days 0, 3, 6, 9, and 12.

Tumor length and width were measured twice per week using a digital caliper, and tumor volumes (V, mm^3^) were calculated using the formula: 0.5×(length×width^2^). Mice were euthanized when the tumor volume reached 2,000 mm^3^. The survival of tumor-bearing mice was monitored and analyzed using Kaplan-Meier curves.

### Flow cytometry analysis

The mice were sacrificed three days after the last treatment. Tumor tissues were collected and cut into pieces. The single cell suspension was prepared by using dissociation buffer (1 mg/mL collagenase IV, 1 mg/ml hyaluronidase, and 20 U/mL DNase in phosphate buffer saline (PBS) plus 2% FBS). The red blood cells were removed by incubating with ACK lysis buffer (CS0001, Leagene). The single cell suspension was stained with Zombie NIR™ Fixable Viability Kit (423105, BioLegend) and immune cell markers (Table S1). Flow cytometry analysis was performed on an LSR Fortessa flow cytometer (BD Biosciences), and data were processed using FlowJo v10 software (TreeStar Inc., USA).

### Single cell sequencing

The single cell suspension was prepared as described above and CD45 positive cells were isolated using CD45 microBeads (130-110-618, Miltenyi). Three samples from the same group were incubated with different hashtag antibodies (TotalSeq-C0301, C0302, C0303, Biolegend), washed and mixed in equal numbers. The single-cell sequencing was performed using Chromium Next GEM Single Cell 3ʹ Reagent Kits v3.1 (10x Genomics) following the manufacturer's protocol. Sequencing was performed on NovaSeq 6000 (Illumina).

### Identification of cell types and cluster marker genes in Seurat

Gene barcode matrices were imported into the Seurat (version 4.0.5) pipeline for quality control and downstream analysis 25. The HTODemux function in Seurat, using its default parameters, was used to assign each individual cell back to its original sample based on the normalized cell hashtag counts. Low-quality cells (< 100 genes/cell, > 5,000 genes/cell, > 20,000 UMIs/cell, > 8% mitochondria) were excluded. The data were then normalized, and highly variable genes were detected using the FindVariableFeatures function. Principal component analysis was performed to reduce the dimensionality of the scRNA-Seq dataset. The top 71 principal components, which explained 90% of the variance in the dataset, were used to perform the downstream analysis. The Harmony package (version 1.0) was used to perform the batch correction 26. Cell clusters were identified using the FindClusters function in Seurat with a resolution of 0.7. They were then visualized using t-distributed stochastic neighbor embedding plots. Each cell cluster was manually categorized into a known biological cell type.

The FindMarkers function in Seurat was used to identify cluster-specific marker genes or differentially expressed genes in an individual cluster. The average expression of the genes within each cluster was calculated, and heatmaps of the average expression of genes were generated using the R package ComplexHeatmap (version 2.8.0).

### Pathway enrichment analysis

Gene set enrichment analysis (GSEA) was performed using WebGestalt 27 to identify the enriched pathways annotated by Gene Ontology, KEGG, and Reactome databases. Pathway size was limited to 500 genes per pathway, and genes were ranked by log 2-fold change calculated by the Findmarkers function in Seurat where 1,000 permutations were used to estimate the FDR for the GSEA analysis.

### Cell communication network construction

The R package CellChat (version 1.1.3) was used to predict the potential cell interactions between the annotated cell clusters after the Seurat analysis 28. Two CellChat objects for the PBS and the OV-mOX40L treatment groups were generated by the createCellChat function using the Seurat object. Cell communication networks were constructed by the computeCommunProb, computeCommunProbPathway, and aggregate net functions. Interaction comparison analysis was performed by the compareInteractions and RankNet function to detect the differentially increased cell interactions in the OV-mOX40L treatment group. The netAnalysis_signalingRole_heatmap and netVisual_aggregate functions were applied to generate the heatmap and circle plots of the interactions among specific cell clusters in each treatment group.

### Statistical analysis

Quantitative data were presented as the mean ± standard deviation (SD) of at least three independent experiments. Data were analyzed using one-way analysis of variance (ANOVA) with Dunnett test. Animal survival was plotted using Kaplan-Meier curves and compared using the log-rank test. A p-value less than 0.05 was considered to be statistically significant (*p < 0.05, **p < 0.01, ***p < 0.001, and ****p < 0.0001).

## Results

### Generation and characterization of OX40L-armed oncolytic HSV-1

**Figure 1:**
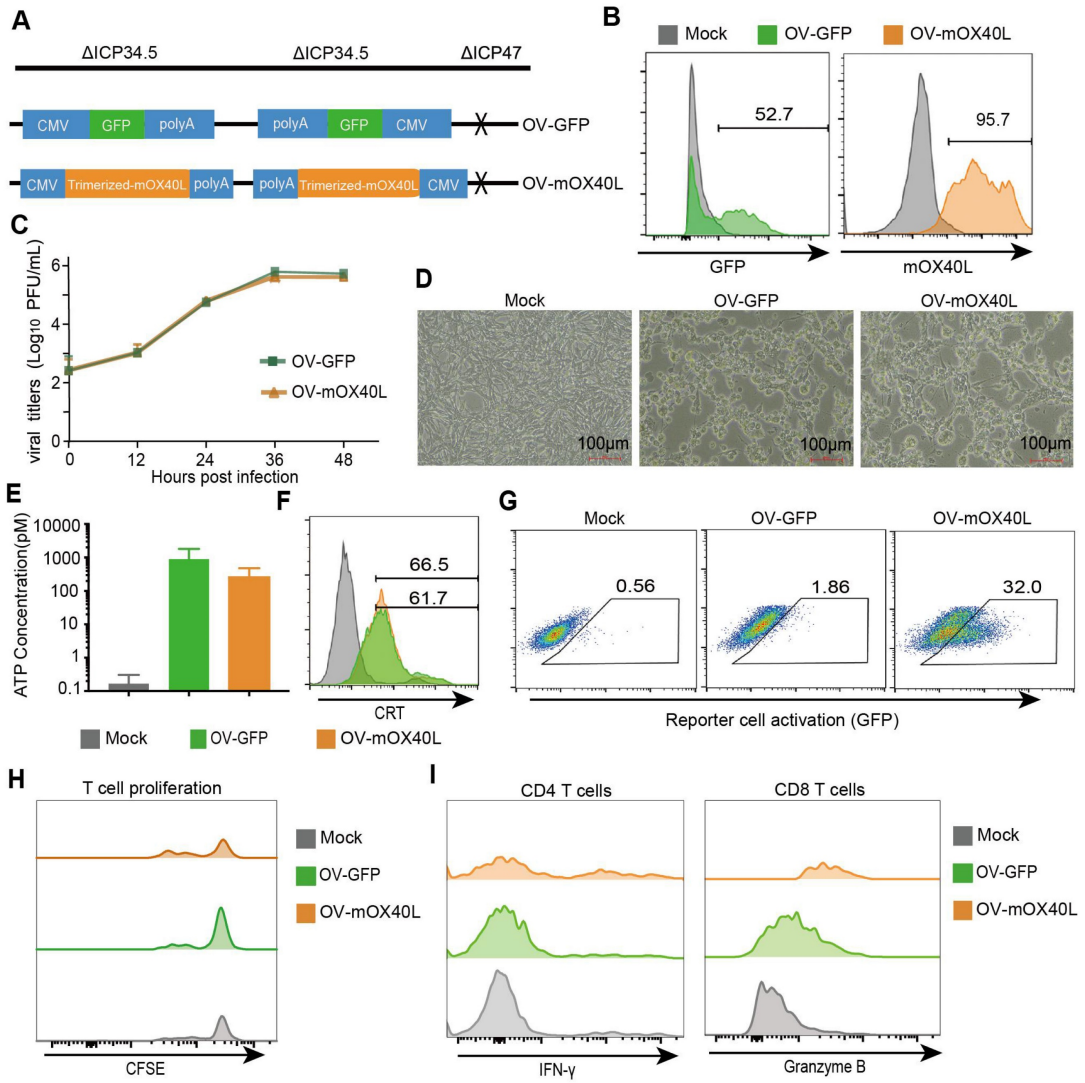
** Generation and characterization of OX40L-armed oncolytic HSV-1. A)** The schematic representation of HSV-1 based oncolytic virus encoding murine OX40L or GFP. The trimerized mOX40L was constructed by IL2 signal sequence-Traf2 coiled-coil domain-mOX40L extracellular domain-PDGFR transmembrane domain. **B)** GFP or membrane-displayed OX40L expression level on KPC cells after infection with OV-GFP or OV-mOX40L. KPC cells were infected with OV-GFP or OV-mOX40L at an MOI of 1. Cells were stained with OX40L antibody after 24 hours and detected by flow cytometry. **C)** Virus replication in the KPC cells. KPC cells were infected with OV-GFP or OV-mOX40L at an MOI of 0.1, and viral titers were determined using plaque assays at different times post infection. **D)** Cytopathic effect (CPE) of OV-mOX40L against KPC cells. KPC cells were infected with OV-GFP or OV-mOX40L at an MOI of 1. Cells were observed under the microscope after 24 hours. **E-F)** ICD of tumor cells was induced by OV-mOX40L. KPC cells were infected with OV-GFP or OV-mOX40L for 48 hours. The released ATP was detected by the ATP detection kit (E) and the cell surface exposure of calreticulin was analyzed by flow cytometry (F). The data for ATP concentration were representative of three independent experiments. **G)** Effect of OV-mOX40L on Jurkat-NF-κB-GFP-OX40 reporter cells. HEK293FT cells were infected with OV-GFP or OV-mOX40L and cocultured with Jurkat-NF-κB-GFP-OX40 reporter cells, which express GFP upon activation of the OX40-OX40L signaling pathway. The GFP expression of the reporter cells was analyzed by flow cytometry. **H)** Effect of OV-mOX40L on T cell proliferation. CFSE-labeled T cells were cocultured with OV-infected KPC cells in 96-well plate precoated with anti-CD3 antibody for three days. The proliferation of T cells, which exhibited decreased CFSE fluorescence intensity, was detected by flow cytometry. **I)** Effect of OV-mOX40L on T cell activation. T cells were cocultured with OV-infected KPC cells in 48-well plate precoated with anti-CD3 antibody for 24 hours. The activation markers IFN-γ and granzyme B of T cells were detected by flow cytometry.

Considering that TRAF2-derived coiled-coil domain could stabilize OX40L trimerization 29, we constructed trimerized mOX40L consisting of the IL2 signal sequence, Traf2 coiled-coil domain, OX40L extracellular domain, and PDGFR transmembrane domain. We evaluated the agonistic potential of trimerized mOX40L and whole natural mOX40L using Jurkat-NF-κB-GFP-OX40 reporter cells, which express GFP upon activation of the OX40-OX40L signaling pathway. Trimerized mOX40L-transfected HEK293FT showed slightly elevated reporter cell activation compared to natural mOX40L-transfected HEK293FT cells (Figure S1). Therefore, we utilized trimerized mOX40L for the oncolytic virus modification. To generate OV-mOX40L, the trimerized mOX40L was inserted into the backbone of *ICP34.5* and *ICP47* double-deleted oncolytic HSV-1 (Figure 1A). Vero cells were infected with OV-mOX40L and membrane-displayed OX40L was determined by flow cytometry (Figure 1B). The pancreatic cancer cell line KPC was infected with either OV-GFP or OV-mOX40L, and the modification of the OV did not attenuate their replication capability (Figure 1C) and tumoricidal activity (Figure 1D). Induction of immunogenic cell death (ICD) was measured by the release of ATP and surface CRT expression according to previous studies 30-33. Although the release of ATP increased after oncolytic virus infection, the difference between OV-mOX40L and OV-GFP infection was minor (Figure 1E). The surface expression of CRT also increased after infection with OV-mOX40L (Figure 1F). In addition, the function of OV-mOX40L was assessed using Jurkat-NF-κB-GFP-OX40 reporter cells. In co-culture with the Jurkat-based reporter cells, the OV-mOX40L pre-infected HEK293FT cells stimulated the reporter cells more than the OV-GFP pre-infected HEK293FT cells (Figure 1G). To further evaluate the function of OV-mOX40L on T cells, we conducted *ex vivo* assays by isolating mouse splenic T cells and co-culturing them with OV-infected KPC cells. The CFSE assay showed that OV-mOX40L-infected KPC cells promoted the proliferation of T cells (Figure 1H). Furthermore, in comparison to OV-GFP-infected KPC cells, OV-mOX40L-infected KPC cells significantly increased the expression levels of IFN-γ and granzyme B (GZMB) in CD4+ T cells and CD8+ T cells, respectively (Figure 1I). These results indicated that OV-mOX40L treatment activated both CD4+ and CD8+ T cells. Additionally, coculturing Tregs with OV-mOX40L-infected KPC cells slightly downregulated the expression of Foxp3 on Tregs (Figure S2).

### OV-mOX40L inhibited tumor growth in a syngeneic pancreatic cancer mouse model

**Figure 2:**
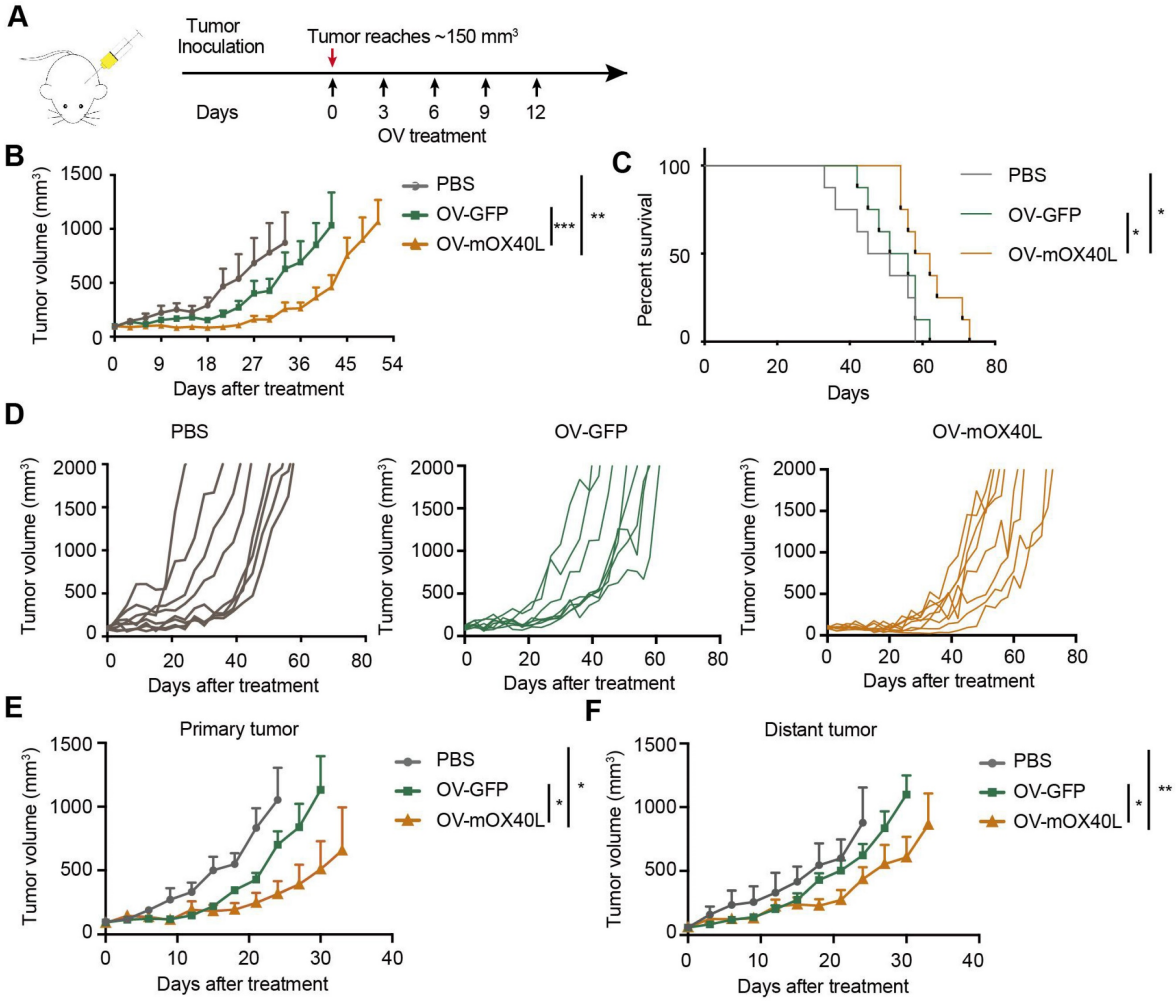
** Anti-tumor effect of OV-mOX40L in syngeneic KPC pancreatic cancer model. A)** Schematic of the regimen of oncolytic virus treatment. KPC were subcutaneously implanted into the flank of C57BL/6J mice. When the tumor size reached about 150 mm^3^ (set as day 0; n=8 per group), the tumor-bearing mice were intratumorally injected with 2×10^6^ PFU of OV-GFP or OV-mOX40L per mouse on day 0, 3, 6, 9, and 12. **B)** The tumor growth curves were shown until they exceeded 1,000 mm^3^. The tumor volumes were measured twice every week. **C)** The Kaplan-Meier survival analysis is shown. Log-rank test was performed for statistical comparison of survival curves. **D)** The individual tumor growth curve of PBS, OV-GFP and OV-mOX40L groups. **E and F)** Anti-tumor activity of OV-mOX40L in bilateral KPC-bearing mouse model. KPC cells were implanted subcutaneously into left and right flanks of mice, respectively. PBS, OV-GFP or OV-mOX40L was intratumorally injected into right side tumors, and the growth of the injected primary tumor (E) and the non-injected distant tumor (F) was shown.

A syngeneic mouse model was used to assess the inhibitory effects of OV-mOX40L on pancreatic cancer growth. KPC cells were inoculated into C57BL6/J mice, which demonstrated intense desmoplasia and an immunosuppressive environment that mimicked the TME of PDAC patients. Tumor-bearing mice were treated with OV-GFP or OV-mOX40L (Figure 2A). Intratumoral administration of OV-mOX40L delayed the tumor growth rate and prolonged the survival of mice, with a median survival time of 60 days in the OV-mOX40L group and 53.5 days in the OV-GFP group, respectively (Figure 2B-D).

Furthermore, we examined whether OV-mOX40L inhibited tumor growth at local and abscopal sites using a bilateral tumor-bearing mouse model. KPC tumor cells were implanted in both flanks of mice, and tumors on the right side were treated with OV-mOX40L, OV-GFP, or PBS and monitored for 35 days. In this experiment, both primary and distant tumor growth were reduced in the OV-mOX40L group compared to the OV-GFP group, indicating that virotherapy induced a systemic antitumor immune response (Figure 2E and F).

### Local OV-mOX40L treatment reinvigorated intratumoral immune cells

To explore the mechanism underlying OV-mOX40L treatment, tumor-infiltrating immune cells and cancer-associated fibroblasts (CAFs) were analyzed three days after the last treatment using flow cytometry.

**Figure 3:**
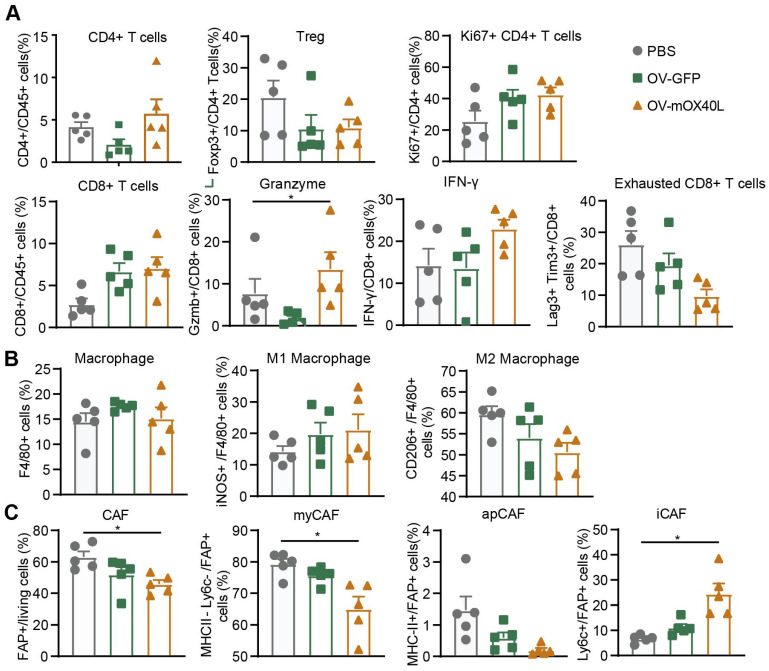
** Analysis of tumor-infiltrating immune cells and fibroblasts after the treatment with oncolytic viruses.** Mice were subcutaneously implanted with KPC cells and treated with oncolytic viruses or PBS. Tumor tissues were collected 3 days after the last treatment. **A-C)** The single-cell suspension was prepared from tumor, stained with the fluorescent antibodies and subjected to flow cytometry analysis for the profiling of T cells (A), macrophages (B) and fibroblasts (C). Statistical significance was determined by ANOVA tests and multiple comparisons.

Treatment with OV-mOX40L reduced Treg counts and increased the proportion of proliferated CD4 T cells. Furthermore, the proportion of CD8+ T cells among CD45+ immune cells and the expression of IFN-γ and granzyme B (GZMB) in CD8+ T cells were moderately upregulated, and the number of exhausted CD8+ T cells with PD-1 and LAG-3 expression decreased (Figure 3A and Figure S3A). This indicated the activation of CD8+ T cells and mitigation of CD8+ T cell exhaustion upon OV-mOX40L treatment.

Upon OV-mOX40L treatment, the number of tumor-associated macrophages (TAM), marked as CD11b+ F4/80+, did not change (Figure 3B and Figure S3B). The pro-inflammatory M1-like macrophages (inducible nitric oxide synthase, iNOS+) increased, whereas the anti-inflammatory M2-like macrophages (CD206+) decreased. The results revealed a shift in the potential of macrophages from anti-inflammatory to pro-inflammatory.

Flow cytometry showed that the number of fibroblast active protein (FAP)-expressing CAFs was significantly decreased by OV-mOX40L treatment. Anti-Ly6C and anti-MHCII antibodies were used to segregate FAP-positive CAF into three populations, including Ly6C-positive inflammatory CAFs (iCAF), MHCII-positive antigen-presenting CAFs (apCAFs) and MHCII/Ly6C-double negative myofibroblastic CAFs (myCAFs). The proportion of myCAFs and apCAFs over total CAF slightly decreased, whereas that of iCAF significantly increased (Figure 3C and Figure S3C).

### OV-mOX40L treatment activated conventional CD4+ T cells and CD8+ cytotoxic T cells and reduced Treg proportion

**Figure 4:**
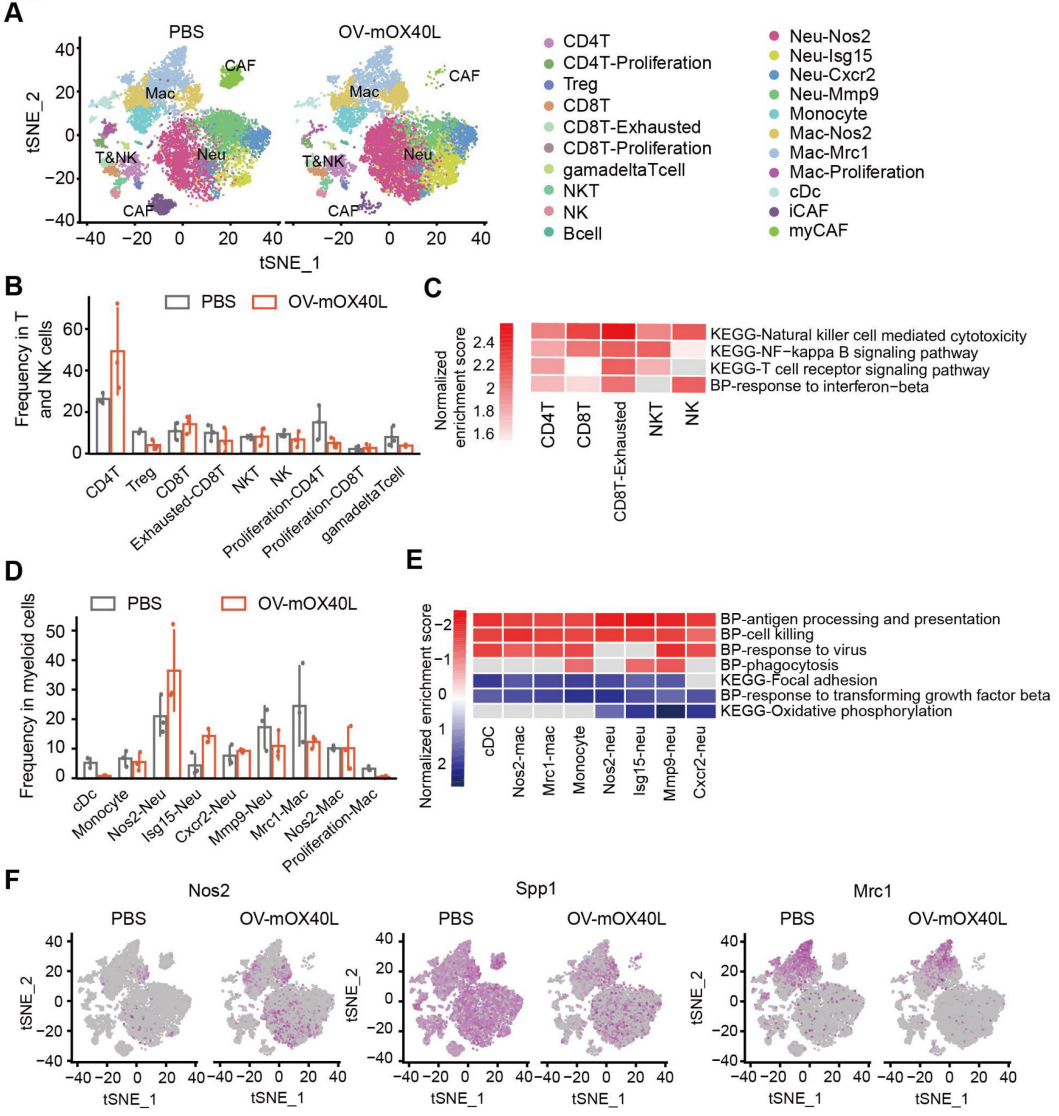
** Single-cell RNA sequencing analysis of the tumor-infiltrating immune cells after OV-mOX40L treatment. A)** All tumor-infiltrating immune cells from PBS and OV-mOX40L treated mice were clustered and showed as tSNE plots. Tumor-infiltrating immune cells were isolated from 3 mice per group and labeled with hashtag antibodies, then mixed in equal numbers for single-cell RNA sequencing. **B)** The proportions of intratumoral T and NK cells in the PBS and OV-mOX40L groups. **C)** GSEA pathway enrichment of OV-mOX40L versus PBS in individual CD8+ T cell, CD4+ T cell, NK and NKT cell clusters. **D)** The proportions of myeloid cells in the PBS and OV-mOX40L groups. **E)** GSEA pathway enrichment of OV-mOX40L versus PBS in individual neutrophils, macrophages and cDC clusters. **F)** Expression of pro-inflammatory marker gene (Nos2) and immunosuppressive marker genes (Spp1 and Mrc1) in the tSNE plot.

To further understand the immune cell populations associated with the anti-tumor response following OV-mOX40L treatment, tumor-infiltrating immune cells were subjected to scRNA-seq analysis. Twenty-one clusters were identified (Figure 4A) and characterized using a series of marker genes (Figure S4A).

Total T and NK cells included clusters of CD8+, CD4+, Treg, NK, NKT, and γδ T cells (Figure 4B). CD4+ T cells were defined as *cd3d* and* cd4* positive cells. Treg cluster displayed a high expression of *Foxp3*, and proliferating CD4+ T displayed a high expression of *Mki67*. OV-mOX40L treatment significantly increased the proportion of conventional CD4+ T cells and reduced the Treg cell proportion (Figure 4B). CD8+ T clusters were defined as *Cd3d*, *Cd8a* double positive. Two CD8+ T clusters exhibited heterogeneous gene profiles: exhausted CD8+ T cluster (*Pdcd1* and *Lag3*) and proliferating CD8+ T cells (*Mki67*). Following OV-mOX40L treatment, the proportion of activated CD8+ cytotoxic T lymphocytes (CTL) slightly increased, whereas the proportion of exhausted CD8+ T cells dramatically decreased (Figure 4B). Pathway analysis revealed the upregulation of signaling pathways associated with cytotoxicity and T cell activation in both the cytotoxic T cluster and the exhausted CD8+ T cluster upon OV-mOX40L treatment (Figure 4C and Figure S4B). The NK cluster was defined based on the expression of *Nkg7* and *Klrk1*, while the NKT cluster was defined based on the expression of *Ly6c2*, *Cd3d*, *Nkg7*, and *Klrk1* (Figure S4A) 34. Although the relative percentages of NK and NKT cells were moderately reduced by OV-mOX40L treatment (Figure 4B), pathway analysis revealed that both clusters upregulated the NK cell-mediated cytotoxicity pathway upon OV-mOX40L treatment (Figure 4C). Overall, these results indicated that CD4+ T, CD8+ T, and NK cells were activated by OV-mOX40L treatment.

### OV-mOX40L treatment switched the tumor immune microenvironment to a more pro-inflammatory state

Myeloid cells, including DC, monocytes, neutrophils, and macrophages, were identified (Figure 4D). Neutrophils were defined based on the expression of *S100a8* and *S100a9* (Figure S4A). Neu-Nos2 and Neu-Isg15 were defined as N1-like neutrophils with anti-tumor activity owing to their high expression of *Nos2*, *Isg15*, or *Icam1* 35-37, whereas Neu-Cxcr2 and Neu-Mmp9 were defined as N2-like neutrophils with pro-tumor activity and characterized by the expression of *Cxcr2* or *Mmp9* 38, 39. OV-mOX40L treatment increased the proportion of N1-like neutrophils and decreased that of N2-like Neu-Mmp9 (Figure 4D). Treatment with OV-mOX40L induced antigen processing and presentation, cell killing, phagocytosis, and response to virus signal pathway, it also downregulated and focal adhesion pathway in neutrophils (Figure 4E and Figure S4C). Moreover, neutrophils upregulated the expression of *Nos2*, which could contribute to their tumor-suppressive activity (Figure 4F).

Macrophages were defined by the high RNA expression of *Csf1r* and *Itgam*, while monocytes were defined by *Ly6c2* and *Itgam's* high expression and low expression of *Itgax*. The following three macrophage clusters exhibited heterogeneous gene profiles (Figure S4A). Nos2-Mac displayed high expression levels of *Itgam* and* Nos2*. Mrc1-Mac displayed high expression of *Adgre1* and *Mrc1*, and the gene signature of proliferation-Mac was similar to that of Mrc1-Mac, except for the high expression of *Mki67*, indicating that both clusters were immunosuppressive 40, 41. Pathway analysis results revealed that macrophages were more pro-inflammatory after OV-mOX40L treatment than after vehicle control administration (Figure 4E and Figure S4C). Moreover, OV-mOX40L treatment increased IFN-γ expression and induced macrophages to express higher levels of pro-inflammatory marker genes, such as *Nos2* and reduced the expression of immunosuppressive marker genes, such as *Spp1 and Mrc1* (Figure S4D and Figure 4F).

Two clusters of CAFs were identified based on the expression of *Mfap2* (Figure S4A). The myCAF cells were characterized by high expression of collagens and contractile myofibroblast factors, such as *Acta2, Tagln*, and *Col7a1* 42. The iCAF cluster was characterized by the expression of chemokines such as *Cxcl12*. Treatment with OV-mOX40L increased iCAF levels and decreased myCAF levels (Figure S4E).

### Analysis of cell-cell communications in pancreatic cancer

**Figure 5:**
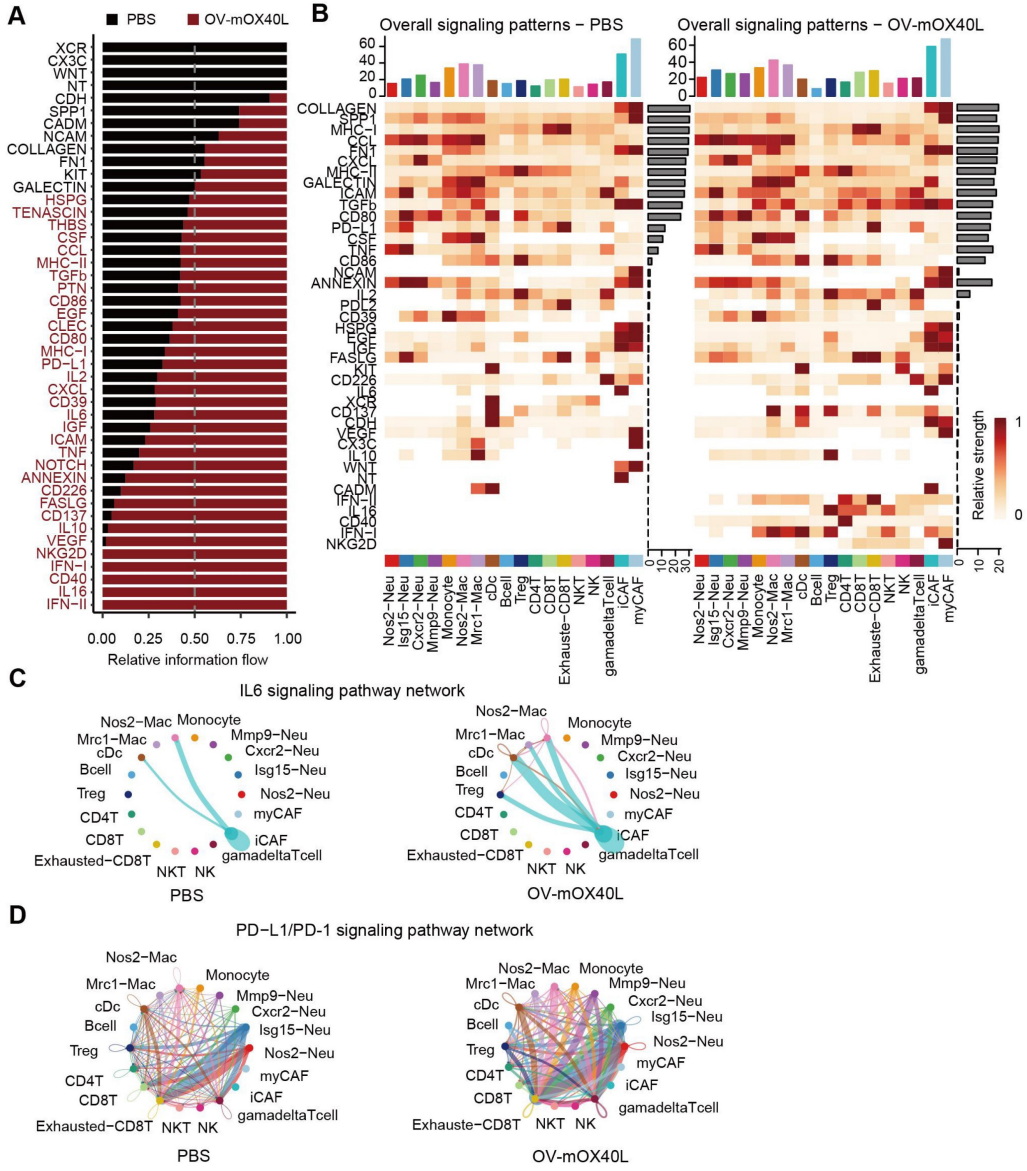
** Identification of major signaling changes after the OV-mOX40L therapy. A)** Normalized interactions were summed as the overall information flow and significantly different signaling pathways were identified between the PBS and OV-mOX40L groups. **B)** The overall signaling patterns among our defined cell clusters in the PBS group and the OV-mOX40L group. **C and D)** The inferred IL6:IL6R/gp130 (C) and PDCD1: PD-L1 (D) signaling network among the cell populations represented by the nodes. The edge width represents the interaction strength of the specific pathway.

Unbiased cell-cell interaction inference analysis was performed across all immune cell types using CellChat to dissect global alterations 28, 43. We analyzed the signaling pathways with the highest combined interaction strength ratios in the PBS and OV-mOX40L groups (Figure 5A). The signaling communication pathways of Cd226: Nectin2/Pvr, Tnf: Tnfrsf1a/Tnfrsf1b, TGFb: TGFbR/ACVR1, VEGF: Flt1/Flt4/Kdr, PDCD1: PD-L1 and IL6: IL6R/gp130, which were related to the poor prognosis, were upregulated with OV-mOX40L treatment, suggesting that these pathways could be potential targets for combination therapy with OV-mOX40L. The overall signal pattern of the clusters was further analyzed. After treatment with OV-mOX40L, interactions of the PDCD1: PD-L1 signaling pathway were observed in a variety of cell types, including T cells, macrophages, and neutrophils, whereas the IL6:IL6R/gp130 pathway was primarily observed in CAFs and macrophages (Figure 5B). Then, we focused on the PDCD1: PD-L1 and IL6:IL6R/gp130 pathways.

Intercellular IL6 signaling interactions among macrophages, DCs, Tregs, and iCAFs were present in the OV-mOX40L group, but only macrophage-iCAF interactions were observed in the PBS group (Figure 5C). Moreover, PD-L1 pathway interactions among immune cells were enhanced by OV-mOX40L treatment (Figure 5D). The upregulation of IL6 44-46 and PD-L1 47 pathways could negatively impact anti-tumor immunity, providing the rationale for combining OV-mOX40L with neutralizing antibodies against IL6 or PD-L1 pathways for the treatment of pancreatic cancer.

### The combination therapy of OV-mOX40L with anti-IL6 and anti-PD-1 antibodies prolonged the survival of PDAC-bearing mice

**Figure 6:**
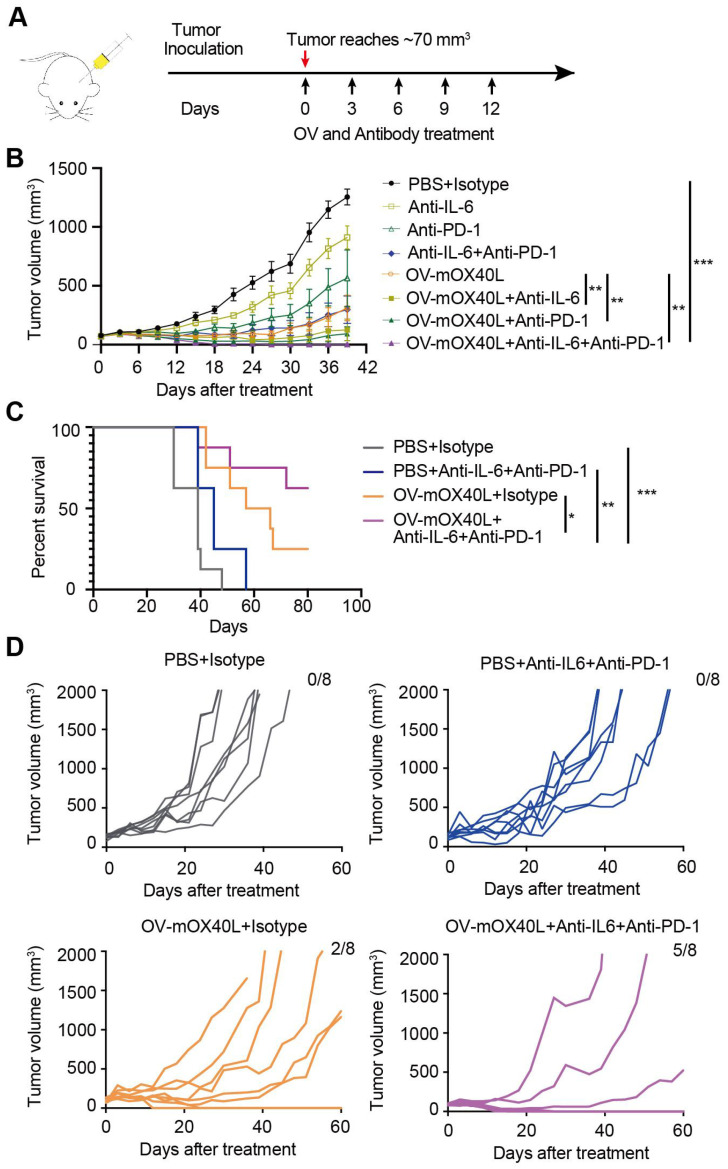
** The combination therapy of OV-mOX40L with anti-IL6 and anti-PD-1 antibodies prolonged the survival of PDAC-bearing mice. A)** Schematic of the regimen of oncolytic virus treatment. KPC were subcutaneously implanted into the flank of C57BL/6J mice. When the tumor size reached about 70 mm^3^ (set as day 0; n=7 per group), the tumor-bearing mice were randomly divided into 8 groups and intratumorally injected with 2×10^6^ PFU OV-mOX40L or 100 μL PBS per mouse on day 0, 3, 6, 9, and 12. 100 μg of each antibody present in each group was intraperitoneally injected per mouse on days 0, 3, 6, 9, and 12. **B)** The tumor volumes were measured every third day and presented by the tumor growth curve (n=7). **C)** Kaplan-Meier survival analysis of KPC-bearing mice is shown. KPC were subcutaneously implanted into the flank of C57BL/6J mice. When the tumor size reached about 100 mm^3^ (set as day 0; n=8 per group), the tumor-bearing mice were randomly divided into 4 groups and intratumorally injected with 2×10^6^ PFU OV-mOX40L or 100 μL PBS per mouse on days 0, 3, 6, 9, and 12. 100 μg of each antibody present in each group was intraperitoneally injected per mouse on day 0, 3, 6, 9, and 12. Log-rank test was performed for statistical comparison of survival curves. **D)** The tumor volumes were measured every 3 days and presented by the tumor growth curve (n=8).

We combined neutralizing antibodies against IL6 or PD-1 in OV-mOX40L to treat a mouse PDAC model (Figure 6A). Compared to OV-mOX40L or neutralizing antibody treatment alone, the combination therapy of OV-mOX40L with anti-IL6 or anti-PD-1 antibody significantly delayed tumor growth, and the most effective tumor inhibition was observed with the triple therapy (OV-mOX40L+anti-IL6+anti-PD-1) (Figure 6B). In a separate experiment, the survival of KPC-bearing mice was monitored. Median survival days in the OV-mOX40L and neutralizing antibody groups were extended from 39 to 61.5 and 45 days, respectively, compared to the basic control (PBS) group, while median survival was not achieved in the triple therapy group as 62.5% of the mice had been cured (Figure 6C and D). This finding corroborated that neutralizing antibodies against IL6 and PD-1 could enhance the effect of OV-mOX40L therapy.

## Discussion

In this study, a novel oncolytic HSV-1 expressing mOX40L was developed for the treatment of pancreatic cancer. This approach was designed to combine the direct oncolytic effect of OV with the immune-stimulating activity mediated by both OV and OX40L. Correspondingly, OV-mOX40L could effectively delay tumor growth and extend survival time in a syngeneic KPC mouse model compared to the parental oncolytic virus.

Oncolytic viruses expressing various kinds of cytokines and immune checkpoints (ICs) have been evaluated in previous studies 48-50. For example, IL2-encoding oncolytic HSV (G47Δ-mIL12) significantly reduced the primary tumor volume and metastasis of triple-negative breast cancer (TNBC) 51. Other studies have revealed that repeated administration of CD40L-armed HSV-1 could improve the survival of PDAC-bearing mice and offer long-term protection from tumor relapse 14. Therefore, OV can be used as a delivery platform and has broad clinical application perspectives. OX40, which mainly induces the activation and proliferation of T cells, has been considered a promising candidate target for cancer immunotherapy. It was reported that OX40 agonistic antibody could improve the survival of the glioblastoma mouse model and showed efficacy in clinical trials 52, 53. Herein, we reported a modified HSV-1 characterized by the delivery of the immunostimulatory factor OX40L, which triggered OX40-OX40L signaling pathway-mediated responses. We engineered OV-mOX40L for PDAC treatment by combining the tumor-targeting effect of OV with the immune-potentiating effect of OX40L.

According to the results of our single-cell transcriptomic analysis and flow cytometry of tumor-infiltrating immune cells, OV-mOX40L treatment decreased Foxp3+ Tregs, activated CD4+ and CD8+ T cells, and resulted in less exhausted CTLs. T cell activation mediated by OX40-OX40L interaction showed increased release of inflammatory cytokines such as IFN-γ, a crucial factor that polarizes macrophages toward the M1 phenotype 54. Under the synergistic or additive action of OV and OX40L, M2-like macrophages and protumor N2-like neutrophils showed reduced proportion and boosted anti-tumor immune responses, whereas pro-inflammatory N1-like neutrophils were remarkably increased after OV-mOX40L treatment. Thus, this study demonstrated that OV-mOX40L treatment was able to convert the immunosuppressive TME towards a more immunoreactive state and establish a more supportive immune niche to activate endogenous T cells.

Stromal fibroblasts play a critical role in supporting tumor growth and drive immunosuppression. Thus, it is important to investigate the effect of OV on the stromal components of pancreatic cancer. Notably, this KPC model mimicked the intense extracellular matrix characteristic of pancreatic cancer. Additionally, flow cytometry results revealed that OV-mOX40L treatment significantly decreased CAFs.

Currently, the efficacy of an isolated strategy targeting stromal desmoplasia or immune cells has been met with limited success for the treatment of pancreatic cancer. These results illustrate the complexity of the pancreatic cancer TME and suggest that combining complementary stroma-targeted and immune-cell-targeted modalities is a rational strategy. Our study confirmed the multifaceted role of the mOX40L-armed oncolytic virus in remodeling the desmoplasia and immunosuppressive tumor immune microenvironment of pancreatic cancer. Moreover, the results from our scRNA-seq analysis revealed that PDCD1: PD-L1 and IL6:IL6R/gp130 signaling interactions were enhanced between CAFs and macrophages as well as between neutrophils and T cells after OV-mOX40L treatment. OV-mOX40L, in combination with anti-IL6 and anti-PD-1, significantly extended the survival of KPC-bearing mice, indicating that combination therapy targeting CAFs and immune cells has considerable potential for clinical applications.

We also observed that Cd226: Nectin2/Pvr, Tnf: Tnfrsf1a/Tnfrsf1b, TGFb: TGFbR/ACVR1 and VEGF: Flt1/Flt4/Kdr signaling pathways were upregulated upon OV-mOX40L treatment. Activation of these pathways could reduce the antitumor immune response; therefore, targeting them could potentially enhance the efficacy of OV-mOX40L. Actually, the power of scRNA-seq could guide the design of different combination therapy. For example, Gulhati *et al*. designed triple therapy with anti-4-1BB/anti-LAG3/CXCR1/2 inhibitor based on high-dimensional immune profiling and the combination therapy resulted in a durable and complete response 55.

In conclusion, the combination of the tumor-targeting OV and the immune-potentiating OX40L has shown promising results for the treatment of pancreatic cancer. OX40L-armed OV therapy can boost T-cell response and potentiate anti-tumor efficacy by remodeling the TME. The combination of OV-mOX40L and anti-IL6/anti-PD-1 antibodies remarkably prolonged the survival of PDAC-bearing mice. These findings provided important evidence for the efficacy and mechanisms of OV-mOX40L treatment and may therefore contribute to monotherapy or combination therapy of OV-mOX40L for PDAC treatment.

## Supplementary Material

Supplementary figures and table.

## Abbreviations

PDAC: pancreatic ductal adenocarcinoma
OV: Oncolytic virus
HSV-1: Herpes simplex virus-1
TME: tumor microenvironment
TAM: tumor-associated macrophages
FAP: fibroblast active protein
CAF: cancer-associated fibroblasts
apCAFs: antigen-presenting CAFs
iCAF: inflammatory CAFs
myCAFs: myofibroblastic CAFs
Tregs: Regulatory T cells
DC: dendritic cells
GSEA: gene set enrichment analysis
GZMB: granzyme B
iNOS: inducible nitric oxide synthase
IFN-γ: interferon-γ
IL-2: interleukin-2
IL-6: interleukin-6
ICD: immunogenic cell death
MOI: multiplicity of infection
SD: standard deviation
ANOVA: one-way analysis of variance
PBS: phosphate buffer saline
PVDF: polyvinylidene fluoride
