# Supplementary material for: OX40L-Armed Oncolytic Virus Boosts T-cell Response and Remodels Tumor Microenvironment for Pancreatic Cancer Treatment
Source: Theranostics. 2023 Jul 9;13(12):4016–29. doi: 10.7150/thno.83495 (PMC10405835; doi:10.7150/thno.83495)
Supplement: Supplementary file 1 — Supplementary figures and table. [file thnov13p4016s1.pdf]

Supplementary Materials

Supplementary Figure and Figure Legends

Supplementary Table

Supplementary Figures and Figure Legends

Figure S1

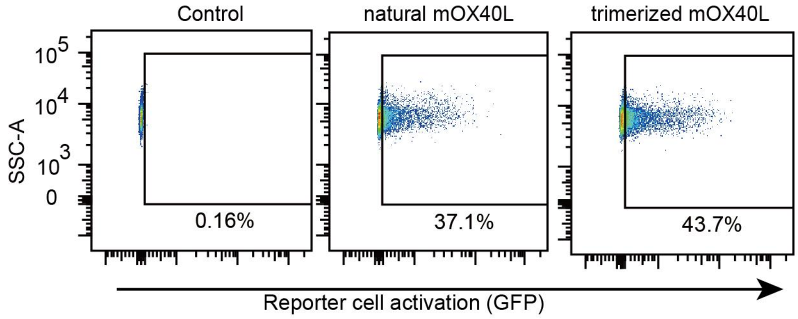

Figure S1. Agonistic activity of the natural mOX40L and trimerized mOX40L. HEK293FT cells were transfected with the whole natural mOX40L or trimerized membrane bound mOX40L and cocultured with Jurkat-NF-κB-GFP-OX40 reporter cells, which express GFP upon activation of the OX40-OX40L signaling pathway. The GFP expression of the reporter cells was analyzed by flow cytometry.

Figure S2

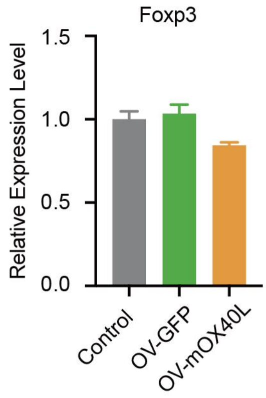

Figure S2. Effect of OV-mOX40L on Foxp3 expression of Tregs. Tregs were cocultured with OV-infected KPC cells in 48-well plate for 24 hours. Foxp3 expression was detected by quantitative real-time PCR.

Figure S3

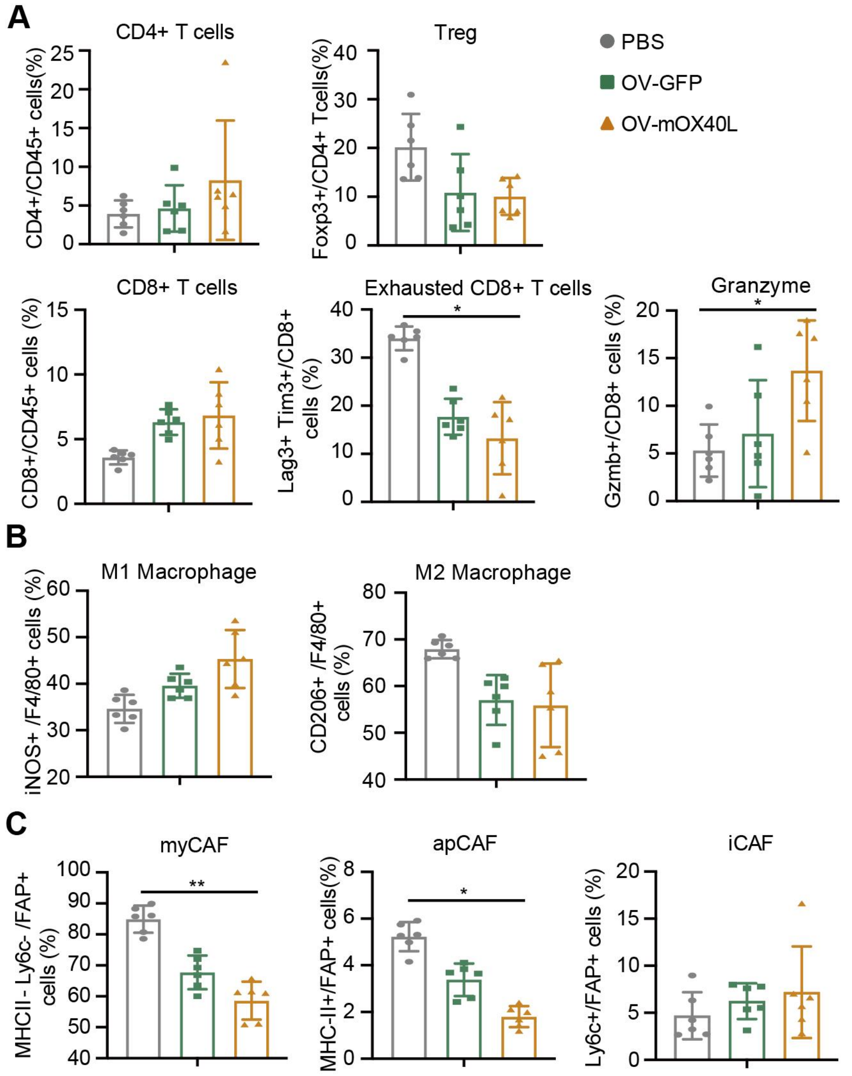

Figure S3. Flow cytometry analysis of tumor-infiltrating immune cells and fibroblasts after the treatment with oncolytic viruses. Mice were subcutaneously implanted with KPC cells and treated with oncolytic viruses or PBS. Tumor tissues were collected 3 days after the last treatment. A-C) The single-cell suspension was prepared from tumor, stained with the fluorescent antibodies and subjected to flow cytometry analysis for the profiling of T cells (A), macrophages (B) and fibroblasts (C). Statistical significance was determined by ANOVA tests and multiple comparisons. Figure S3 was a repeated experiment for Figure 3.

Figure S4

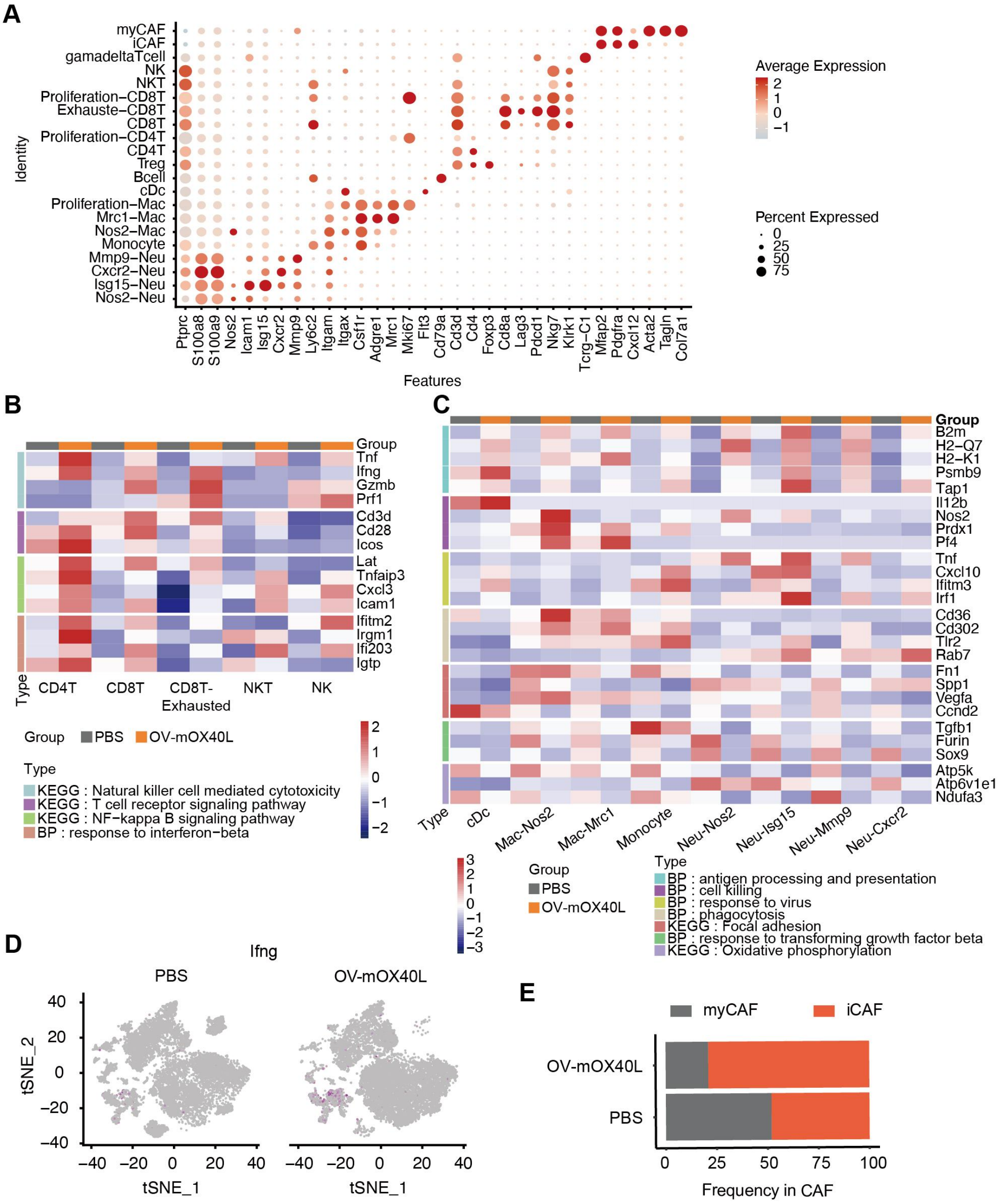

Figure S4. Analysis of intratumoral immune cells by scRNA-seq. A) Bubble plots showed the expression of cell markers in each cluster. **B)** Heatmap displayed the expression of the selected genes in each pathway of lymphocyte cluster. **C)** Heatmap displayed the expression of the selected genes in each pathway of neutrophil, macrophage and cDC cluster. **D)** The expression of IFN-γ from PBS and OV-mOX40L group was shown by the t-SNE plot. **E)** The proportions of CAF clusters.

Supplementary Table

Table S1. List of anti-mouse antibodies for flow cytometry analysis

Cell marker	Catalog number	Vendor	Fluorescence
CD45	103107	biolegend	FITC
CD4	100428	biolegend	Pacific Blue™
CD8a	100752	biolegend	Brilliant Violet 510™
CD366 (Tim-3)	134006	biolegend	Alexa Fluor® 647
CD223 (LAG-3)	125226	biolegend	PE/Cyanine7
CD279 (PD-1)	135228	biolegend	PE/Dazzle™ 594
Ki-67	151210	biolegend	PE
IFN-γ	505846	biolegend	PE/Dazzle™ 594
FOXP3	320014	biolegend	Alexa Fluor® 647
Granzyme B	372208	biolegend	PE
CD11b	101224	biolegend	Pacific Blue™
I-A/I-E (MHCII)	107614	biolegend	APC
F4/80	123149	biolegend	Brilliant Violet 650™
CD206 (MMR)	141732	biolegend	PE/Dazzle™ 594
Nos2 (iNOS)	696806	biolegend	PE
Ly-6C	128033	biolegend	Brilliant Violet 510™
FAP	bs-5758R-AF647	Bioss	Alexa Fluor® 647
